# Primary malignant chest wall tumors: analysis of 40 patients

**DOI:** 10.1186/1749-8090-9-106

**Published:** 2014-06-19

**Authors:** Reza Bagheri, Seyed Ziaollah Haghi, Mahmoud reza Kalantari, Alireza Sharifian Attar, Maryam Salehi, Azadeh Tabari, Maliheh Soudaneh

**Affiliations:** 1 Cardio-Thoracic Surgery & Transplant Research Center, Emam Reza hospital, Faculty of medicine, Mashhad University of Medical Sciences, Mashhad, Iran; 2 Endoscopic & Minimally Invasive Surgery Research Center, Department of Anesthesiology, Ghaem Hospital, Faculty of Medicine, Mashhad University of Medical Sciences, Mashhad, Iran; 3 Solid Tumor Treatment Research Center, Omid Hospital, Faculty of medicine, Department of community medicine, Mashhad University of Medical Sciences, Mashhad, Iran; 4 Mashhad University of Medical Sciences, Mashhad, Iran; 5 Mashhad University of Medical Sciences, Mashhad, Iran

**Keywords:** Primary chest wall tumors, Resection and reconstruction, Survey

## Abstract

**Background:**

Primary chest wall tumors originate from different constructions of thoracic wall. We report our multidisciplinary experience on primary thoracic tumor resection and thoracic reconstruction, the need to additional therapy and evaluating prognostic factors affecting survival.

**Methods:**

We performed a retrospective review of our prospectively maintained database of 40 patients treated for malignant primary chest wall tumor from 1989 to 2009. Patients were evaluated in terms of age, sex, clinical presentation, type of imaging, tissue diagnosis methods, pathology, surgical technique, early complications, hospital mortality, prevalence of recurrence and distant metastases, additional treatment, 3 years survival and factors affecting survival.

**Results:**

Male/Female (F/M) = 1, with median age of 43.72 years. Mass was the most common symptoms and the soft tissue sarcoma was the most common pathology. Resection without reconstruction was performed in 5 patients and Thirty-five patients (87.5%) had extensive resection and reconstruction with rotatory muscular flap, prosthetic mesh and/or cement. Overall, 12.5% (5/40) of patients received neoadjuvant therapy and 75% (30/40) of patients were treated with adjuvant therapy. The 3-year survival rate was 65%. Recurrences occurred in 24 patients (60%), 14 developed local recurrences, and 10 developed distant metastases. The primary treatment modality for both local and distant recurrences was surgical resection; among them, 10 underwent repeated resection, 9 adjuvant therapy and 5 were treated with lung metastasectomy. The most common site of distant metastasis was lung (n = 7). Factors that affected survival were type of pathology and evidence of distant metastasis.

**Conclusion:**

Surgery with wide margin is the safe and good technique for treatment of primary chest wall tumors with acceptable morbidity and mortality.

## Background

Primary chest wall tumors involve a wide various groups of soft tissue and skeletal structures of thorax. They originate from different constructions of the thoracic wall and may occur in 2 main tissue types: single and combined. Owing to the few number of such lesions, diagnosis and appropriate therapeutic approach is needed and further evaluations should be considered. There are various ways to diagnose these tumors including: fine needle aspiration and biopsy (FNA & B), incisional and excisional biopsy. Chest wall tumors have been classified according to these criteria: cell type, involved tissue, sensitivity to radiation and benign against malignant [1].

Malignant primary tumors are mostly symptomless and grow slowly. With the extension of these masses, pains occur. Chest wall resection due to diverse etiologies can cause extensive chest wall defects which may involve soft and skeletal tissues. There are 2 ways to cover deep chest wall defects: prosthetic or biologic mesh and/or soft tissue flaps with excellent blood supply [2,3]. Surgeons are not always able to close the defect with antilogous tissue, in the tumors which are too large and are given adequate margins [4].

Titanium implants in combination with synthetic or biologic mesh can be a safe and effective way of reconstructing large full thickness chest wall defects. Reconstruction varies according to the origin of the tumor: mesh and cement is used to reconstruct tumors originating from bone tissue, while and in soft tissue sarcomas muscular flap is administered [3].

The aim of this study is to evaluate the outcomes of surgical treatment in malignant primary chest wall tumors, and the factors affecting survival.

## Methods

Between 1989 and 2009, this case-series study was performed on 40 patients with primary chest wall tumors, who underwent chest wall reconstruction at Qaem and Omid Hospital of Mashhad University of Medical Sciences-Iran. Follow-up period was 3 years.

Inclusive criteria: 1) malignant primary chest wall tumor with definitive pathologic diagnosis 2) performing appropriate surgery 3) three years follow up after surgery.

Exclusive criteria: 1) Other types of chest wall tumors (benign or metastatic) 2) inadequate surgical treatment 3) less than three years follow up.

Patients were evaluated in terms of: age, sex, clinical presentation, type of imaging, tissue diagnosis methods, pathology, surgical technique, early complications, hospital mortality, prevalence of recurrence and distant metastases, additional treatment, 3 years survival and factors affecting survival. This article is approved in regional ethic committee of Mashhad University of Medical Sciences (project number: 89844).

### Statistical analysis

All data were analyzed using SPSS software. Kruskal-Wallis test, Mann-Whitney test and Chi-Square were applied. P-Value < 0.05 was statistically significant.

## Result

**Figure 1:**
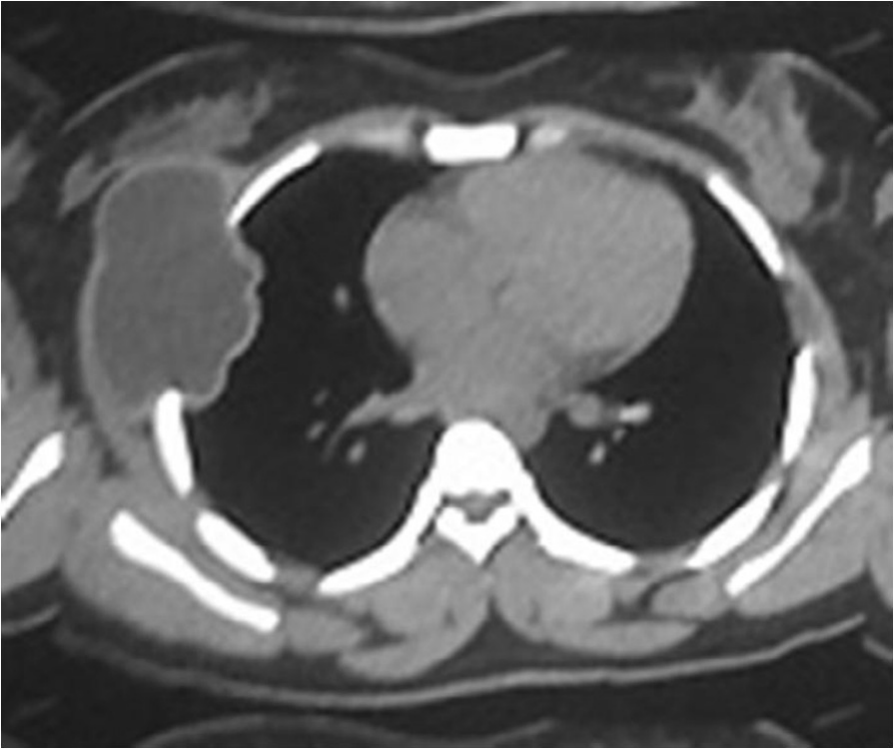
CT scan of a patient with chest wall tumor.

There were 20 men and 20 women, aged 11 to 86 years, with a median age of 43.72 years. Clinical presentations were: palpable mass (97.5%, of them, 20% were tender), fever (5%), and cough (17.5%). Chest X-ray and Computed tomography was performed for all patients; however, MRI was done in only 15 patients (37.5%) to assess neurovascular involvement. Figure 1 showed CT scan of patients with chest wall tumors.

There were 3 diagnostic procedures; fine needle aspiration and biopsy (FNA & B) was performed in all patients, of them, it was diagnostic in 5 patients (12.5%). Hence, tissue samples were obtained by 2 other methods in 35 patients. Incisional biopsy in 8 patients with tumor more than 4cm in size (20%) and excisional biopsy in 27 cases with tumor less than 4cm (67.5%). According to pathology results, patients were classified into 6 groups: soft-tissue sarcoma (18 patients, 45%), chondrosarcoma (12 patients, 30%), osteosarcoma (6 patients, 15%), small round cell tumor (4 patients, 10%), plasmacytoma (4 patients, 10%), giant cell tumor (1 patient, 2.5%). In our series, 18 out of 40 primary chest wall tumors were soft tissue sarcoma. Soft tissue sarcoma was the most common primary chest wall tumor, and MFH was the most common pathology among soft sarcomas (33%, and 15% of total tumors).

**Figure 2:**
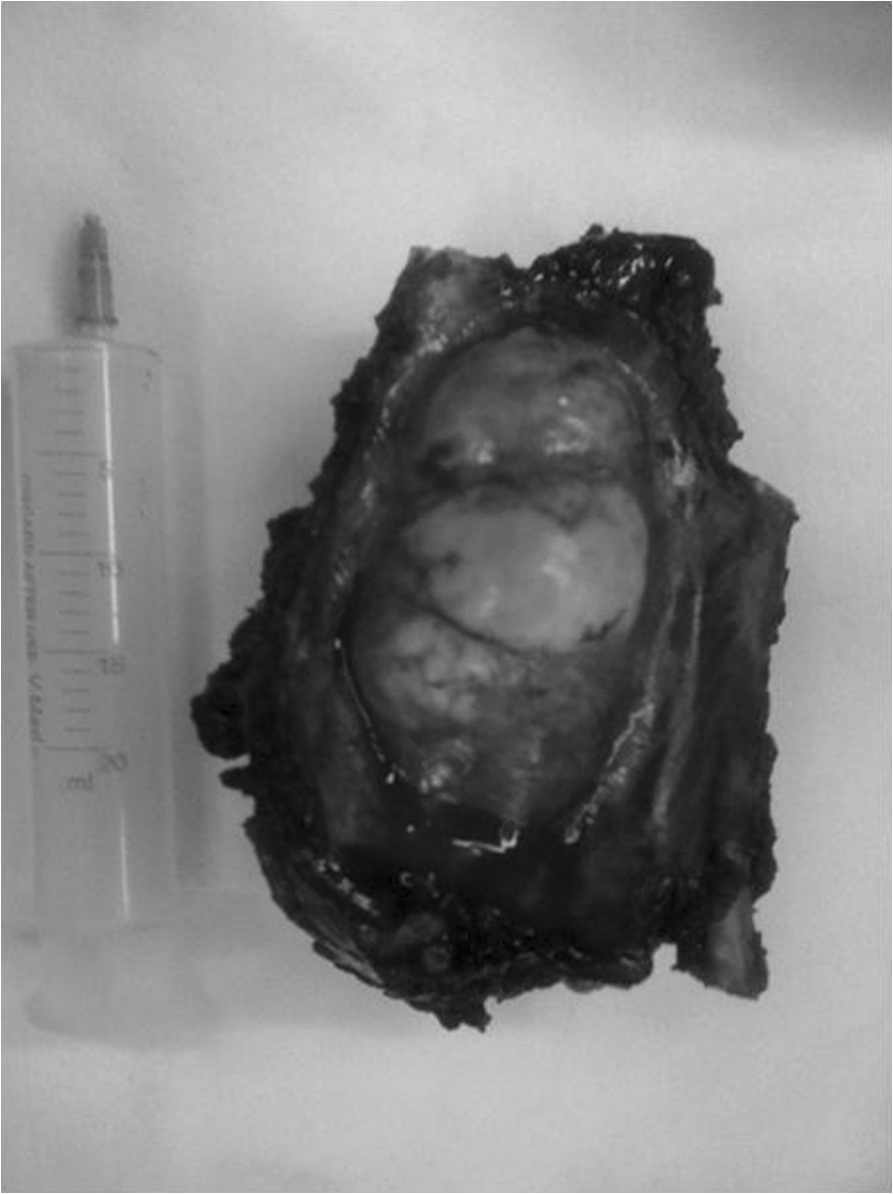
Resected specimen of patient with chest wall tumor.

**Figure 3:**
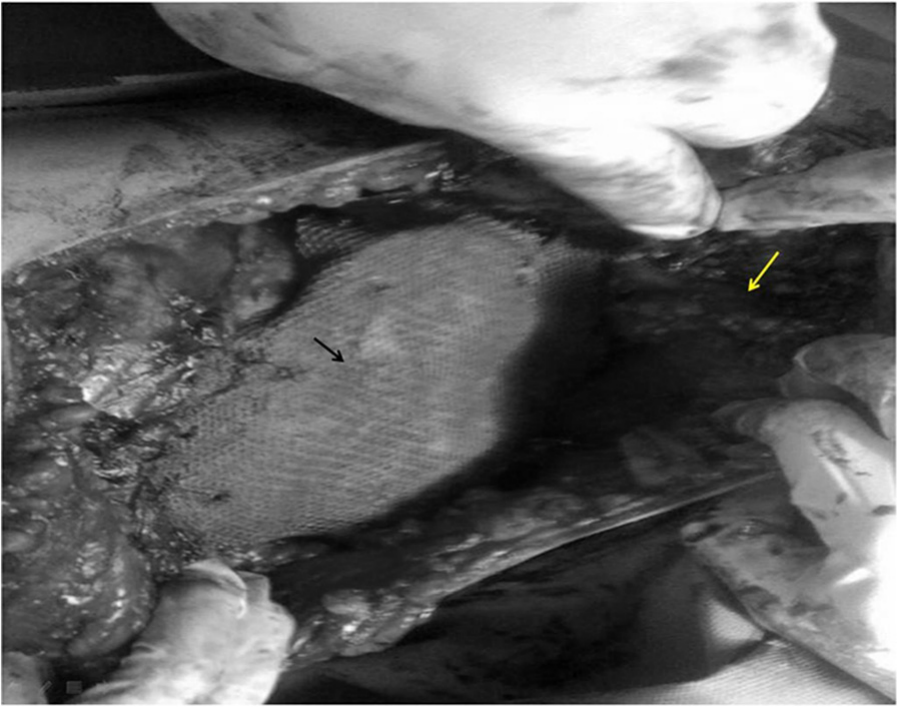
**Technique of reconstruction.** Black arrow: Meshed and cement, White arrow: Latissimus dorsi muscle flap.

**Figure 4:**
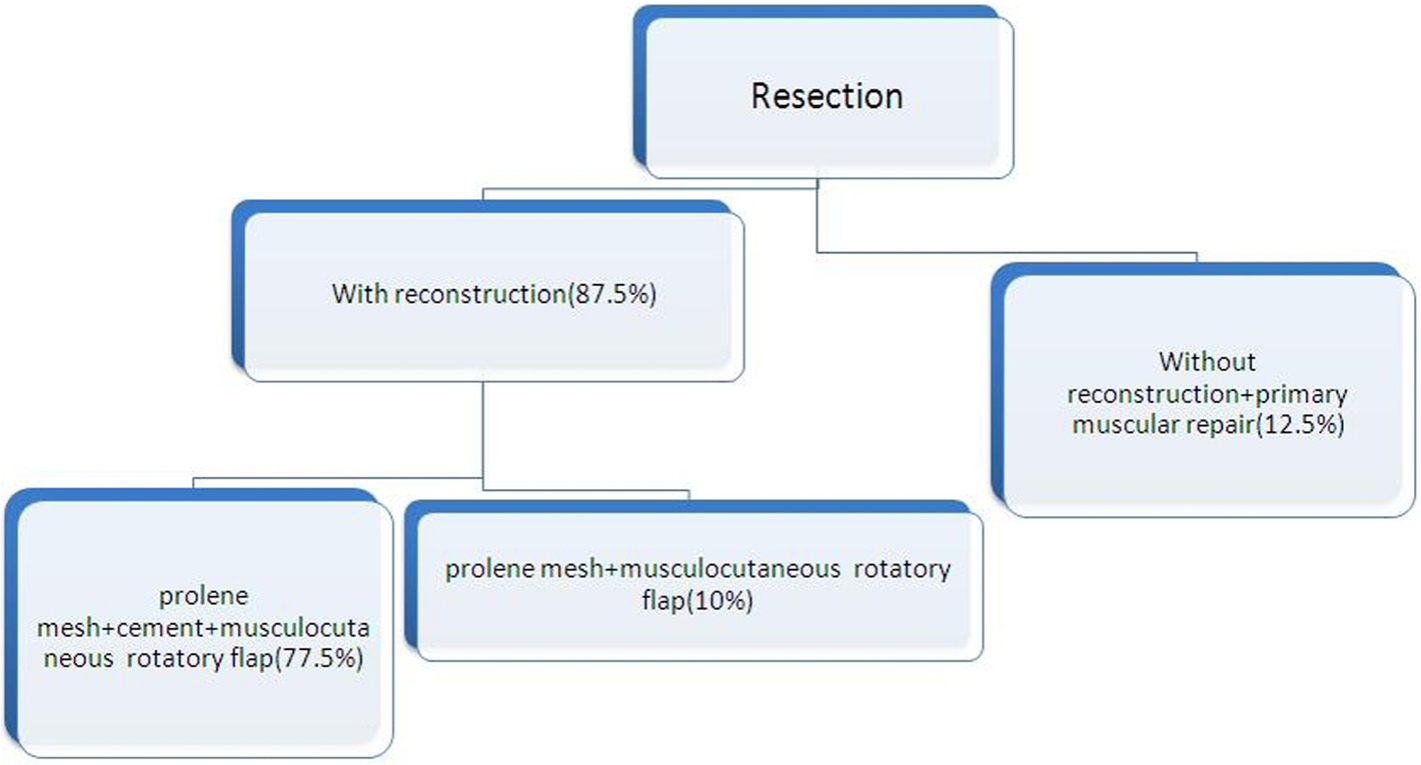
Type of surgery in our patient.

**Figure 5:**
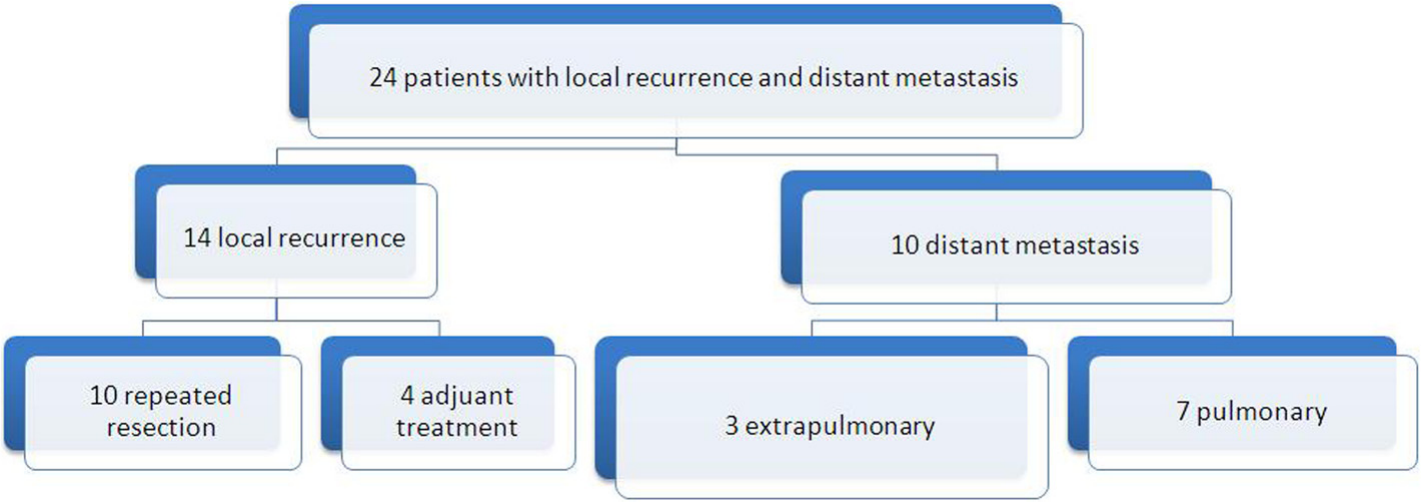
Distant metastases and local recurrence Figure.

35 out of 40 tumors (87.5%) arise from both soft tissue and adjacent ribs and other 5 tumors (12.5%) originated from sternum. Adequate margin of resection was 4^cm^. Moreover, reconstruction was required in anterior tumors sized more than 5^cm^ and posterior tumors more than 10^cm^. five patients underwent resection without reconstruction: 2 with Low grade malignant fibrous histiocytoma (MFH), 1 with costal Chondrosarcoma, 1 with Liomyosarcoma and 1 Fibrosarcoma.Resection was performed in 4 men and 1 woman. Moreover, extensive resection and reconstruction was done in 19 women and 16 men. Figure 1 and Figure 2 showed resected specimen and the use of mesh and cement in combination with muscle flap in reconstruction.Latissimus dorsi flap was the common muscle flap used in our work (30 patients). In five patients with sternal resection bilateral pectoral muscle flap was used (Figure 3). Figure 4 shows technique in surgical resection. Early post operative complications were seen in 8 patients: 4 patients with wound infection (10%), 2 with sarcoma (5%) and in 2 other patients atelectasia occured (5%). All of these 8 patients were treated with medical therapy. Hospital mortality was 0% (defined as death during 30 days after operation). Prevalence of local recurrence and distant metastasectomy are detailed in Figure 5.

**Figure 6:**
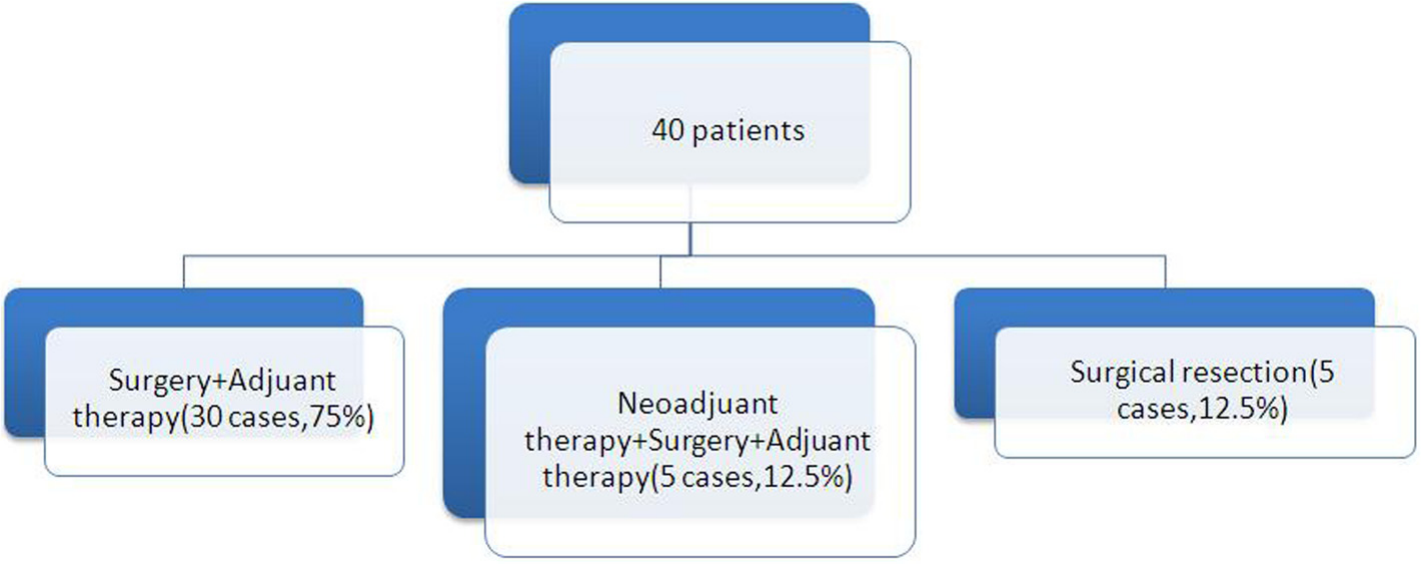
Need for adjuvant therapy.

Extra pulmonary metastases originated differently: bone (2.5%), liver (2.5%), retroperitoneal (2.5%). These 3 patients with extra pulmonary metastasis underwent adjuvant therapy.2 different approaches administered in 7 patients with pulmonary metastasis: metastasectomy and adjuvant therapy were performed in 5 and 2 patients respectively (12.5% and 5%). The need for adjuvant therapy showed in Figure 6.

3 years survival was 65%. 14 patients died during follow up, of these, 8 were men and 6 were women. The most mortal pathologies were: round cell tumor (4 cases), soft tissue sarcoma and osteochondroma (3 patients each), plasmacytoma (2 cases), osteosarcoma and chondrosarcoma (1 patient each). Table 1 shows factors that affected survival of our patients.

**Table 1:** This table shows the prognostic factors affecting in survival

**Factors affecting survival**	**Survey [N(%)]**		**P.value**
	**Good**	**Bad**	
Age (mean ± SD)	44.80 ± 18.3	41.71 ± 19.7	0.62
Sex			
Male	12 (30)	14 (35)	0.50
Female	8 (20)	6 (15)	
Histopathology			
Soft sarcoma	15 (37.5)	3 (7.5)	0.03
Osteosarcoma + chondrosarcoma	3 (7.5)	1 (2.5)	0.11
Soft sarcoma + chondrosarcoma	0	1 (2.5)	0.35
Chondrosarcoma	7 (17.5)	5 (12.5)	0.72
Osteosarcoma	2 (5)	4 (10)	0.16
Round cell	0	4 (10)	0.01
Plasmacytoma	2 (5)	2 (5)	0.60
Giant cell tumor	1 (2.5)	0	0.65
Technique of surgery			
Resection	5 (12.5)	0	0.14
Resection and reconstruction	21 (52.5)	14 (35)	
Distant metastasis			
Yes	0	26 (65)	0.001
No	10 (25)	4 (10)	

Our purpose is to discuss the role of operative technique and tissue pathology in survival. In this study, we report our multidisciplinary experience with primary chest wall sarcomas that included induction (adjuvant and neoadjuvant) therapy. 3 years disease-free survival was estimated 65 %( 26 patients). Patients with round cell tumor and soft sarcoma have high mortality rate and also distant metastasis is one of the main factors affecting survival (P_value: 0.011, 0.028 and 0.00 respectively).

## Discussion

Primary chest wall tumors are not common and are presented with different symptoms. Patients usually get symptomatic with palpable mass, pain or both at the site of the tumor [1,5].

They may arise from diverse structure of the thoracic wall [6].

Reconstruction of the chest wall is a reliable efficient management in patients with chest wall tumors. Other treatment alternating reconstruction surgeries are as follows: 1-various types of flaps (skin, muscle, myocutaneous, transferred ones from another location and free flaps); 2- to close the defect primarily [7].

In 2011, a study by Guo L et al. revealed that single or combined flaps are useful to repair soft tissue defect. In addition, synthetic materials should be applied in severe and extensive defect to reconstruct thoracic skeleton [8].

In a study performed on 11 cases with primary thoracic sarcoma, D’Alessandro P at al. cited that a safe and effective one stage surgical procedure for various chest wall defects is tumor resection and reconstruction using prosthetic mesh [9].

Also, in 2012, Berthet JP et al. discussed that titanium implants in combination with strong synthetic or biologic mesh in a one-stage procedure can safely use for reconstructing major chest wall defects [3].

A retrospective study performed on 5 patients described the use of biologic mesh as a safe and dependable alternative to synthetic prostheses and tissue repair in pediatric chest wall reconstruction [10].

Between 2005 and 2009, Bosc R and colleagues performed post-resection reconstruction in 22 patients with primary or metastatic chest wall tumors. They concluded that full-thickness chest wall resection has prolonged palliation and cure in these patients [2].

In 2012, 51 patients with primary chest wall sarcomas underwent full-thickness resection. The majority of high risk sarcomas (including: soft tissue, bony and desmoids tumors) were treated. Kachroo P et al. cited that local and distal recurrence may be decreased by neoadjuvant systemic therapy and improve survival [11].

There are different factors affecting the type of the reconstruction. In 2010, Tepliakov VV et al. pointed out that the extension of the resected site; cosmetic outcomes of the tissue flaps and localization of the tumor are some of the affecting factors. They also cited that the cornerstone in treating thoracic wall tumors is surgical methods [12].

Pathology of primary chest wall tumors obtained by thin sections is too varied and results in different outcomes and survival. A study by James B et al. on 23 patients with malignant tumors revealed that only 13% of tumors of skeletal origin have more than five years survival [1].

In 2011, Girotti P and colleagues performed a study on 101 patients with sternal tumors. They described that local complication rate may be reduced by an adequate sternal resection at the first operation and prosthetic integration with surrounding tissues [13].

A study by Otsuka T and coauthors revealed that in patient with malignant fibrous histiocytoma (MFH) resection and reconstruction surgery has good results [14].

In 2013, a study by McMillan RR et al. on 192 patients who underwent resection for soft tissue sarcoma of the chest wall revealed that, local or distant recurrences of soft tissue sarcomas commonly occurred after surgical resection, but, both can make reasonable results if treated with resection [15].

## Conclusion

Surgery with wide margin is the safe and good technique for treatment of primary chest wall tumors with acceptable morbidity and mortality.

## Abbreviations

F: Female; M: Male; FNA & B: Fine needle aspiration and biopsy; MRI: Magnetic resonance imaging; CT scan: Computed tomography scan; MFH: Malignant fibrous histiocytoma.

## Competing of interests

The authors declare that they have no competing interests.

## Authors’ contributions

RB, carried out data collection, writing and study design. SZH, participated in data collection and study design. MRK, performed Study design and data collections. AS attar, carried out data collections and writing. MS, carried out data statistical analysis. AT, carried out data collections and writing. MS, carried out Study design, data collections and data analysis and writing. All authors read and approved final manuscript.
